# Multi-Center Prototype Feature Distribution Reconstruction for Class-Incremental SAR Target Recognition

**DOI:** 10.3390/s26030979

**Published:** 2026-02-03

**Authors:** Ke Zhang, Bin Wu, Peng Li, Zhi Kang, Lin Zhang

**Affiliations:** School of Electronic Engineering, Xidian University, Xi’an 710071, China; 23023222030@stu.xidian.edu.cn (K.Z.); penglixd@xidian.edu.cn (P.L.); 23021212004@stu.xidian.edu.cn (Z.K.); 23021211239@stu.xidian.edu.cn (L.Z.)

**Keywords:** Class-Incremental Learning, Synthetic Aperture Radar, feature fusion, Attributed Scattering Center

## Abstract

In practical applications of deep learning-based Synthetic Aperture Radar (SAR) Automatic Target Recognition (ATR) systems, new target categories emerge continuously. This requires the systems to learn incrementally—acquiring new knowledge while retaining previously learned information. To mitigate catastrophic forgetting in Class-Incremental Learning (CIL), this paper proposes a CIL method for SAR ATR named Multi-center Prototype Feature Distribution Reconstruction (MPFR). It has two core components. First, a Multi-scale Hybrid Attention feature extractor is designed. Trained via a feature space optimization strategy, it fuses and extracts discriminative features from both SAR amplitude images and Attribute Scattering Center data, while preserving feature space capacity for new classes. Second, each class is represented by multiple prototypes to capture complex feature distributions. Old class knowledge is retained by modeling their feature distributions through parameterized Gaussian diffusion, alleviating feature confusion in incremental phases. Experiments on public SAR datasets show MPFR achieves superior performance compared to existing approaches, including recent SAR-specific CIL methods. Ablation studies validate each component’s contribution, confirming MPFR’s effectiveness in addressing CIL for SAR ATR without storing historical raw data.

## 1. Introduction

Synthetic Aperture Radar (SAR) is an advanced active microwave imaging system. It produces high-resolution two-dimensional terrain imagery and operates independently of weather and lighting conditions. This all-weather, day-and-night Earth observation capability is valuable for continuous monitoring across diverse environments [1]. SAR imaging has been widely adopted in topographic mapping, environmental monitoring, disaster assessment, agricultural management, and resource surveying [2,3].

With the rapid development of artificial intelligence, deep learning-based SAR Automatic Target Recognition (ATR) has become a core technology in remote sensing image interpretation and resource monitoring. These systems are critical in real-world scenarios—disaster response, maritime monitoring, infrastructure inspection, and strategic resource assessment. They automatically and accurately identify vehicles, vessels, buildings, and other targets from SAR imagery, supporting safety and resource management [4,5,6,7,8,9].

However, operational environments and mission requirements evolve dynamically. Conventional SAR ATR systems face a key challenge: new target types emerge constantly, requiring models to adapt without full retraining. Most existing deep learning-based SAR ATR methods use a static learning paradigm, trained on a fixed set of target categories [10,11]. When new classes appear, these systems suffer from catastrophic forgetting—sharp degradation in recognition performance of previously learned classes as new knowledge is added [12,13].

This challenge underscores the pressing need for SAR ATR systems that support continuous learning. There is growing demand for systems that efficiently integrate new target class knowledge without full retraining, while maintaining recognition performance on previously learned classes. This need has driven research into incremental learning methods for SAR ATR.

**Figure 1:**
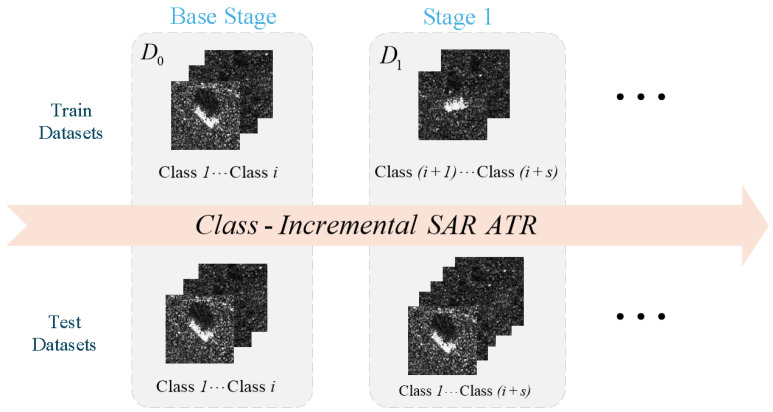
Class-Incremental Learning for Synthetic Aperture Radar Automatic Target Recognition.

Class-Incremental Learning (CIL) provides a structured framework to address catastrophic forgetting in SAR ATR. CIL enables models to learn progressively from a sequence of non-overlapping SAR datasets ($D_{0},D_{1},\ldots ,D_{t}$) that arrive incrementally. The model must acquire new knowledge while maintaining classification accuracy across all previously encountered classes. After each learning phase, the model is evaluated on all learned classes [14], as shown in Figure 1. This reflects real-world usability in evolving operational settings.

CIL methods fall into three main categories: regularization-based, replay-based, and parameter-isolation approaches. Regularization techniques like PASS [15] use prototype augmentation to enhance class discriminability across incremental states. Replay methods such as IL2A [16] leverage class distribution information to replay past class features without storing raw samples.

A recent trend is freezing the feature extractor after initial learning and only incrementally updating the classifier. This avoids storing old-class exemplars and simplifies model updates. Methods like FeTrIL [17] (generating synthetic features), SimpleCIL [18], and FeCAM [19] follow this trend.

**Figure 2:**
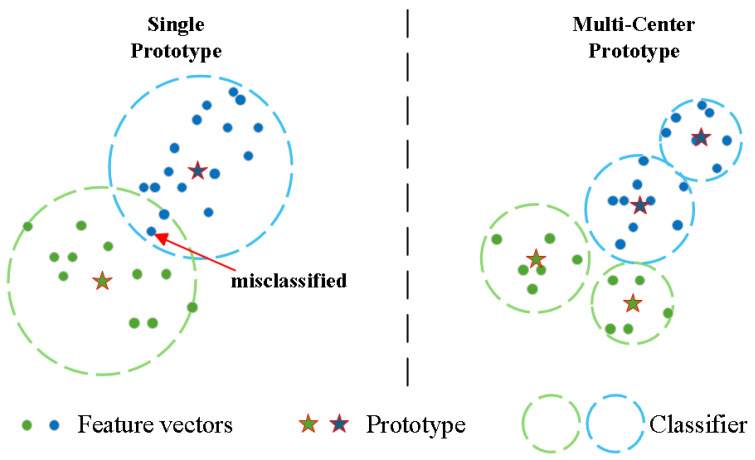
Comparison of single-prototype vs. multi-center prototype representations. Single prototypes fail to capture complex class distributions, leading to feature confusion and classification errors at decision boundaries. Multi-center prototype representation captures intrinsic manifold structure, preserving topological relationships and reducing inter-class interference.

However, many current approaches represent each class with a single prototype centroid. This oversimplification causes problems in SAR image recognition. SAR targets have complex, multi-modal feature distributions due to aspect-dependent scattering and target configuration variations [20]. When new classes are introduced without retraining the feature extractor, their feature distributions often overlap significantly with old classes in the embedding space. The single-prototype representation fails to capture these complex manifolds, leading to severe feature confusion and misclassification (Figure 2).

Although FeTrIL and SimpleCIL streamline the incremental learning pipeline, they cannot handle such complex distributions effectively. Their reliance on global centroids or simple feature generation fails to model intra-class diversity. Thus, SAR target recognition requires methods that finely represent each class’s complex feature distributions.

To address these issues, this paper proposes the Multi-center Prototype Feature Reconstruction (MPFR) method. MPFR differs fundamentally from single-prototype approaches by modeling each class distribution with multiple cluster centroids. This enables faithful reconstruction of complex feature manifolds during incremental phases. Combined with multi-modal SAR feature fusion, MPFR robustly captures the intricate scattering characteristics of radar targets while mitigating catastrophic forgetting.

MPFR operates in two phases: base phase (initial model training and prototype selection) and incremental phases (feature reconstruction and classifier updates). During incremental learning, synthetic features of old classes are generated from stored multi-center prototypes. These are combined with new target class real features to update the classifier. This mitigates catastrophic forgetting while preserving inter-class discrimination, even with SAR noise and high variability.

The main contributions of this paper are threefold:

- Novel CIL Framework: MPFR departs from single-prototype approaches by using multiple prototypes per class. This accurately captures the complex, multi-modal feature distributions of SAR targets caused by aspect-dependent scattering and configuration variations.
- Advanced Multi-modal Feature Fusion: A Multi-scale Hybrid Attention feature extractor fuses SAR amplitude images with physically meaningful Attributed Scattering Center features. A joint supervision strategy optimizes the feature space for incremental learning.
- Comprehensive Experimental Validation: Extensive evaluations on MSTAR [21] and SAR-AIRcraft-1.0 [22] datasets show MPFR achieves state-of-the-art performance. It outperforms existing CIL approaches in average accuracy and forgetting rate. Ablation studies validate each component’s contribution.

## 2. Related Work

### 2.1. SAR Automatic Target Recognition

SAR Automatic Target Recognition (ATR) has evolved significantly, transitioning from traditional model-based approaches to modern deep learning techniques. Early SAR ATR systems predominantly relied on template matching and model-based methods [23,24,25,26]. While these methods offer strong interpretability, they are often too simplistic to handle the complexities of real-world SAR ATR scenarios.

The rise of deep learning has revolutionized SAR ATR, with Convolutional Neural Networks (CNNs) demonstrating substantial performance improvements. Pioneering work by [3] introduced CNN architectures specifically tailored for SAR target classification. Subsequent research explored various network designs, including deeper architectures [9], multi-scale networks [27], attention mechanisms [28,29,30], and pre-trained models such as SARATR-X [31]. More recently, methods incorporating electromagnetic scattering characteristics and feature fusion [20,32,33,34] have made considerable progress in achieving both high recognition accuracy and physical interpretability.

### 2.2. Class-Incremental Learning

Class-Incremental Learning (CIL) is a pivotal domain in continual learning designed to overcome the plasticity–stability dilemma. Early approaches primarily relied on regularization. Methods such as LwF [35] introduced distillation losses to preserve the responses of the old model on new data. However, their performance often degrades significantly as the number of incremental tasks increases.

Replay-based methods constitute another major direction. iCaRL [36] pioneered the use of exemplar replay. WA [37] improved this by aligning weight magnitudes. PODNet [38] enhanced replay efficiency via spatial-based distillation. More recently, methods like IL2A [16] explored feature-level replay to circumvent privacy and storage constraints.

A prominent trend is decoupling feature extraction from classifier learning. Approaches such as FeTrIL [17], SimpleCIL [18], and FeCAM [19] typically freeze the backbone after the initial stage and focus on optimizing the classifier using robust feature representations.

In the broader computer vision domain, significant progress has been made with exemplar-free approaches. A notable method is Adversarial Drift Compensation (ADC) [39]. ADC introduces a mechanism to adversarially perturb current task samples to mimic old class distributions, compensating for semantic drift without storing exemplars. While highly effective on optical datasets (e.g., ImageNet, CIFAR), its application to the complex, speckle-noise-rich SAR domain remains an open challenge, as the generated adversarial perturbations may not fully align with the physical scattering properties of radar targets.

Integrating CIL into SAR ATR introduces unique challenges. Our work focuses on the standard CIL setting where training data for new classes is abundant, but access to historical data is restricted. In this context, the primary challenge is preserving the complex manifold structures of learned classes without overwriting them with new information.

Several domain-specific methods have been proposed to address this. MLAKDN [40] utilizes multilevel adaptive knowledge distillation, while IncSAR [41] employs a dual fusion framework. Furthermore, recent work based on MIM-CIL [42] aims to mitigate forgetting by maximizing the dependency between historical and current representations.

However, despite these advancements, most existing SAR CIL methods still rely on global constraints or assume relatively compact, unimodal class distributions. They often fail to explicitly model the fragmented, multi-modal feature manifolds caused by large viewpoint variations in radar imagery. Our proposed MPFR method addresses this gap by employing a multi-center prototype strategy. Unlike methods based solely on information maximization or distillation, MPFR explicitly reconstructs the diverse geometric modes of target features.

## 3. Proposed Method

This section details the MPFR method. It first presents the overall framework, clarifying the workflow of the base phase and incremental phase. Then it elaborates on three core components: Multi-scale Hybrid Attention-based Feature Fusion (extracting discriminative multi-modal features), Feature Space Optimization (using joint supervision to enhance feature discriminability), and Multi-Center Prototype Feature Distribution Reconstruction (modeling complex class distributions and generating synthetic features). Each component’s design principles and implementation details are explained.

### 3.1. Overall Framework

**Figure 3:**
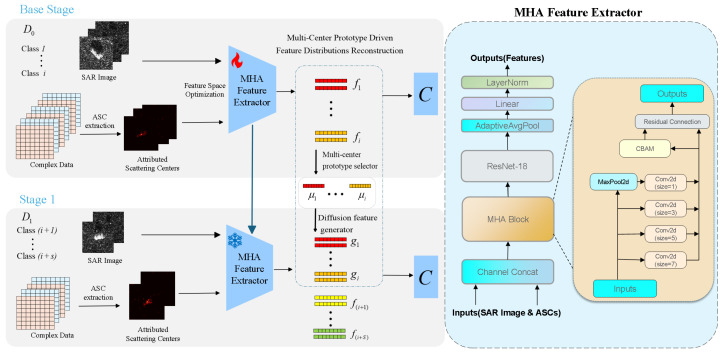
Overall framework of the proposed method, showing the base phase for initial feature extraction and prototype selection, and the incremental phase for feature reconstruction and classifier updating.

The MPFR framework operates in two distinct phases: base phase and incremental phase, as shown in Figure 3. The base phase focuses on training a high-performance feature extractor and selecting multi-center prototypes. The incremental phase generates synthetic old-class features via diffusion and updates the classifier using a hybrid feature space. This balances feature extractor stability and classifier plasticity, addressing catastrophic forgetting.

In the base phase, a gradient-based method [43] extracts Attributed Scattering Centers (ASC) that model targets’ high-frequency electromagnetic scattering characteristics. These ASC features are fused with SAR amplitude images via a feature space optimization strategy to jointly train a multi-scale hybrid attention feature extractor.

The trained feature extractor *F* processes the initial training dataset $D_{0}$ to generate feature vectors $\left\{f_{1},\ldots ,f_{N}\right\}$, which train the initial classifier *C*. A multi-center prototype selector then identifies representative prototypes $\left\{\mu_{1},\ldots ,\mu_{m}\right\}$ for each class and stores them in the prototype set μ.

During incremental learning, the diffusion feature generator synthesizes old-class feature representations $\left\{g_{1},\ldots ,g_{i}\right\}$ using the preserved prototype set μ. These reconstructed features are combined with new target features extracted by the frozen *F* to form a hybrid feature space for training the current incremental classifier *C*. This allows the frozen *F* and updated *C* to recognize all previous targets while adapting to new classes.

After each incremental phase, the multi-center prototype selector identifies new-class prototypes and adds them to μ.

Algorithm 1 formally describes MPFR’s complete class-incremental learning procedure:
**Algorithm 1** Class-Incremental Learning Procedure of MPFR.**Input:** 
Initial dataset $D_{0}$; Incremental datasets $\left\{D_{1},\ldots ,D_{T}\right\}$**Output:** 
Trained feature extractor *F*; Updated classifier *C*; Multi-center prototypes μ  1:**Base Phase Training:**  2:Train feature extractor: $F\leftarrow {\mathrm{Train}}_{\mathrm{FE}}\left(D_{0}\right)$                        ▹ Using the loss in Equation (9)  3:Extract initial features: $F_{0}=F\left(D_{0}\right)$  4:Train initial classifier: $C\leftarrow {\mathrm{Train}}_{\mathrm{CLS}}\left(F_{0}\right)$                                  ▹ e.g., using cross-entropy  5:Select multi-center prototypes: $\mu \leftarrow \mathrm{Kmeans}(F_{0},K=m)$                  ▹*m* clusters per class  6:**Incremental Phase Learning:**  7:**for** phase s=1 **to** *T* **do**  8:      Extract new class features: $F_{s}=F\left(D_{s}\right)$  9:      Generate diffusion features: $G_{\mathrm{old}}\leftarrow \mathrm{DiffusionGenerator}\left(\mu \right)$10:      Construct hybrid feature space: $F_{h}=F_{s}\cup G_{\mathrm{old}}$11:      Update classifier: $C\leftarrow {\mathrm{Train}}_{\mathrm{CLS}}\left(F_{h}\right)$12:      Update prototype set: $\mu \leftarrow \mu \cup \mathrm{Kmeans}(F_{s},K=m)$13:**return** *F*, *C*, μ

### 3.2. Multi-Scale Hybrid Attention-Based Feature Fusion

This subsection details the Multi-scale Hybrid Attention (MHA) feature extractor designed to fuse SAR amplitude images and scattering center features. It follows a systematic workflow which includes the following steps in sequence: scattering center extraction, ASC reconstructed image generation, dual-modal feature extraction, multi-scale encoding, hybrid attention fusion, and backbone refinement, as shown in Figure 3.

Attributed scattering center features are extracted from raw SAR echo data using gradient-based optimization. The process starts with a parameterized scattering center model that accurately represents high-frequency electromagnetic scattering. For each scattering center, attributes like position (x,y), type, amplitude, and frequency-dependent characteristics are estimated by solving the nonlinear optimization problem:

(1) $$\hat{\theta }=\arg\min_{\theta }{∥s\left(f,\phi \right)-\hat{s}\left(f,\phi ;\theta \right)∥}_{2}^{2}$$

Here, s(f,ϕ) is the measured SAR echo data, and $\hat{s}\left(f,\phi ;\theta \right)$ is the simulated echo using ASC model parameters $\theta =\{\left(x_{k},y_{k},A_{k},\alpha_{k},\gamma_{k},\ldots \right)\}$ for all *k* scattering centers. Gradient-based methods in deep learning frameworks ensure efficient convergence.

Extracted ASC parameters are rasterized into feature maps $S\in R^{H\times W\times C}$ via spatial projection. Each scattering center contributes to its corresponding pixel location based on attributes, generating spatially-aligned representations compatible with SAR amplitude images $A\in R^{H\times W\times 1}$.

The fusion mechanism uses a multi-scale architecture with hybrid attention for adaptive feature weighting. The feature extractor processes the concatenated input X=Concat(A,S) through parallel convolutional paths with different receptive fields:

(2) $$\begin{array}{cc} F^{\left(3\right)} & =\mathrm{ReLU}(\mathrm{BN}\left({\mathrm{Conv}}_{3\times 3}\left(X\right)\right)) \end{array}$$

(3) $$\begin{array}{cc} F^{\left(5\right)} & =\mathrm{ReLU}(\mathrm{BN}\left({\mathrm{Conv}}_{5\times 5}\left(X\right)\right)) \end{array}$$

(4) $$\begin{array}{cc} F^{\left(7\right)} & =\mathrm{ReLU}(\mathrm{BN}\left({\mathrm{Conv}}_{7\times 7}\left(X\right)\right)) \end{array}$$

(5) $$\begin{array}{cc} F^{\left(pool\right)} & =\mathrm{ReLU}(\mathrm{BN}\left({\mathrm{Conv}}_{1\times 1}\left(\mathrm{MaxPool}\left(X\right)\right)\right)) \end{array}$$

Multi-scale encoded features are concatenated for a comprehensive representation:

(6) $$F_{\mathrm{encoded}}=\mathrm{Concat}\left(F^{\left(3\right)},F^{\left(5\right)},F^{\left(7\right)},F^{\left(pool\right)}\right)$$

The hybrid attention fusion module [44] refines features adaptively through sequential processing. Channel attention emphasizes informative feature channels:

(7) $$M_{c}\left(F\right)=\sigma \left(\mathrm{MLP}\left(\mathrm{AvgPool}(F)\right)+\mathrm{MLP}\left(\mathrm{MaxPool}(F)\right)\right)$$

Spatial attention addresses misalignment and highlights discriminative regions:

(8) $$M_{s}\left(F\right)=\sigma \left(f^{7\times 7}\left(\mathrm{Concat}\left(\mathrm{AvgPool}(F),\mathrm{MaxPool}(F)\right)\right)\right)$$

Finally, attention-weighted features are refined via a ResNet-18 backbone:

(9) $$F_{\mathrm{refined}}=\mathrm{ResNet}\text{-}18\left(F_{\mathrm{fused}}\right)$$

This approach captures comprehensive information through multi-scale encoding and enables intelligent feature selection via hybrid attention. It leverages complementary advantages of SAR amplitude information and scattering center features, ensuring robust performance in complex electromagnetic environments.

### 3.3. Feature Space Optimization for Backbone Network Training

**Figure 4:**
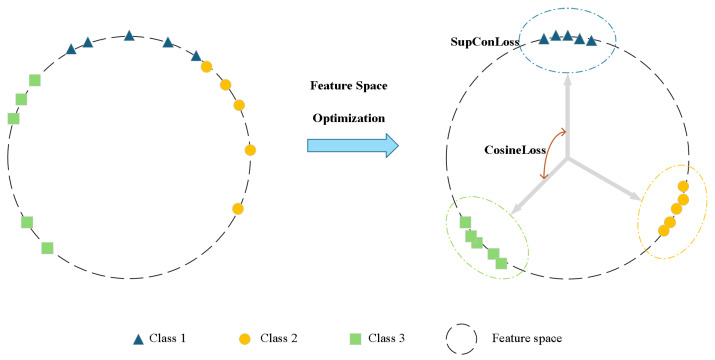
Illustration of the joint supervision strategy for feature space optimization. The framework combines supervised contrastive loss and additive angular margin loss to achieve intra-class compactness and inter-class distinctiveness in the learned feature representations.

To ensure the MHA feature extractor produces discriminative features for incremental learning, a joint supervision strategy combines Supervised Contrastive Loss [45] and Additive Angular Margin Loss [46] (Figure 4). This dual-loss strategy optimizes intra-class compactness and inter-class separability, laying a foundation for subsequent multi-center prototype learning.

Supervised contrastive loss structures the feature space by pulling same-class features together and pushing different-class features apart. For a batch of normalized feature vectors $\hat{z_{i}}=z_{i}/{∥z_{i}∥}_{2}$, the loss is:

(10) $$L_{\mathrm{SupCon}}=-\frac{1}{N}\sum_{i=1}^{N}\frac{1}{\left|P(i)\right|}\sum_{p\in P(i)}\log\frac{\exp(\hat{z_{i}}\;\cdot \;\hat{z_{p}}/\tau )}{\sum_{k\neq i}\exp\left(\hat{z_{i}}\;\cdot \;\hat{z_{k}}/\tau \right)}$$

Here, P(i) is the set of indices for the same class as sample *i* (positive pairs), τ is the temperature hyperparameter, and *N* is the batch size. This guides the model to learn semantically consistent feature embeddings, enhancing robustness.

Additive angular margin loss imposes an angular constraint on the classification layer to improve discriminability. Let $W_{j}$ be the normalized classification layer weight vector, and $\theta_{i,y_{i}}$ be the angle between feature $z_{i}$ and its ground-truth class weight $W_{y_{i}}$. The loss is:

(11) $$L_{\mathrm{Cos}\mathrm{ine}}=-\frac{1}{N}\sum_{i=1}^{N}\log\frac{\exp(s\;\cdot \;\cos\left(\theta_{i,y_{i}}+m\right))}{\sum_{j=1}^{C}\exp\left(s\;\cdot \;\cos\left(\theta_{i,j}\right)\right)}$$

Here, *s* is a scale factor, *m* is the additive angular margin, and *C* is the total number of classes. The angular margin *m* explicitly increases inter-class separation.

The joint training objective combines both losses via weighted summation:

(12) $$L_{\mathrm{Total}}=\lambda_{\mathrm{sc}}L_{\mathrm{SupCon}}+\lambda_{\cos}L_{\mathrm{Cos}\mathrm{ine}}$$

Here, $\lambda_{\mathrm{sc}}$ and $\lambda_{\cos}$ are balancing hyperparameters. This synergy ensures intra-class aggregation via contrastive loss and inter-class separation via angular margin loss, producing powerful fused features with invariance and high discriminability.

### 3.4. Multi-Center Prototype Feature Distribution Reconstruction

**Figure 5:**
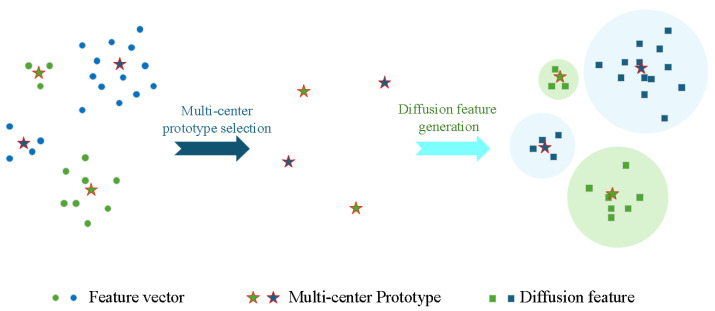
Feature distribution reconstruction for two classes: feature representations of each class are clustered into *m* clusters (visualized here as m=2 for clarity, though experiments use m=10), and the cluster centers are preserved as multi-center prototypes. The left side shows the original feature vectors and multi-center prototypes, while the right side demonstrates the diffusion features generated from these prototypes, reconstructing the feature space distribution for incremental learning.

This subsection presents the core innovation of MPFR: Multi-Center Prototype Feature Distribution Reconstruction. In CIL with a frozen feature extractor, single-prototype class representation causes feature confusion, especially in noisy SAR ATR. MPFR addresses this by capturing complex class distributions through two key steps: multi-center prototype extraction and feature distribution reconstruction, as shown in Figure 5.

For each new class in the current incremental phase, K-means clustering [47] with *m* clusters is applied to extracted feature vectors F. This yields *m* cluster centroids $\mu =\{c_{1},c_{2},\ldots ,c_{m}\}$ as multi-center prototypes. The clustering objective minimizes the within-cluster sum of squares:

(13) $$J=\sum_{i=1}^{n}\min_{j=1}^{m}{∥x_{i}-c_{j}∥}_{2}^{2}$$

Here, $x_{i}$ is a feature vector, and ${∥\;\cdot \;∥}_{2}$ is the Euclidean distance.

It is worth noting that a common alternative to dealing with intra-class diversity is reformulating the problem as subclass classification, where fine-grained labels are treated as mutually exclusive subclasses. However, such an approach relies on the availability of detailed, manually annotated sub-labels, which are often expensive or unavailable in non-cooperative SAR scenarios.

In contrast, our MPFR method utilizes unsupervised clustering to automatically discover these latent multi-modal distributions. By representing each class with *m* adaptive centroids without requiring explicit subclass supervision, MPFR effectively captures the intrinsic geometric structure of the data while avoiding the prohibitive cost of fine-grained annotation. Consequently, multi-center prototypes offer a more practical and robust solution for modeling complex SAR feature distributions than rigid subclass definitions.

During incremental training, old-class feature distributions are reconstructed by generating synthetic features around preserved multi-center prototypes. For each centroid $c_{j}$, $n_{j}$ diffusion features are generated (where $n_{j}$ equals the number of original feature points in the cluster). The standard deviation $\sigma_{j}$ for each cluster is:

(14) $$\sigma_{j}=\sqrt{\frac{1}{n_{j}-1}\sum_{i=1}^{n_{j}}{\left(∥x_{i}-c_{j}{∥}_{2}-{\bar{d}}_{j}\right)}^{2}}$$

Here, ${\bar{d}}_{j}$ is the mean distance from features to centroid $c_{j}$. Diffusion features $G_{j}$ for each centroid are generated as:

(15) $$G_{j}={\left\{v_{i}^{\left(j\right)}|v_{i}^{\left(j\right)}=c_{j}+\epsilon_{i},\;\epsilon_{i}∼N\left(0,\sigma_{j}^{2}I\right)\right\}}_{i=1}^{n_{j}}$$

A class’s complete reconstructed feature space G is the union of all diffusion feature sets:

(16) $$G=G_{1}\cup G_{2}\cup \cdots \cup G_{m}$$

Classifier *C* is trained on a hybrid feature space $F_{h}$ combining old-class reconstructed features and new-class real features:

(17) $$C\leftarrow F_{h}=F_{\mathrm{new}}\cup G_{\mathrm{old}}$$

Here, $F_{\mathrm{new}}$ are new-class feature vectors, and $G_{\mathrm{old}}$ are old-class reconstructed diffusion features. This mitigates catastrophic forgetting while maintaining inter-class discrimination, even with SAR noise and variability.

## 4. Experiments and Results

This section validates MPFR’s effectiveness through comprehensive experiments. It first describes experimental settings (datasets, implementation details, evaluation metrics) to ensure reproducibility. Then it compares MPFR with state-of-the-art CIL methods on two public SAR datasets, analyzing performance advantages. Finally, ablation studies quantify the contribution of each core component, verifying the rationality of the proposed framework.

### 4.1. Datasets and Configurations

#### 4.1.1. MSTAR Dataset

The Moving and Stationary Target Acquisition and Recognition (MSTAR) dataset [21], developed by Sandia National Laboratory, is a widely recognized benchmark in the SAR target recognition field. Operating in the X-band with a resolution of 0.3 m, it encompasses a diverse set of vehicles with variations in configuration, depression angles, and operating conditions, making it a standard for evaluating SAR ATR algorithms. For this study, we adopt the Standard Operating Condition (SOC) scenario with a cross-depression angle protocol: training samples are collected at a 17° depression angle, while testing samples are acquired at 15° to simulate practical scenario differences.

Three incremental learning scenarios with varying difficulty are designed:

- **Setting A**: 6 incremental phases (T=6), 1 new class per phase (S=1)
- **Setting B**: 3 incremental phases (T=3), 2 new classes per phase (S=2)
- **Setting C**: 2 incremental phases (T=2), 3 new classes per phase (S=3)

**Figure 6:**
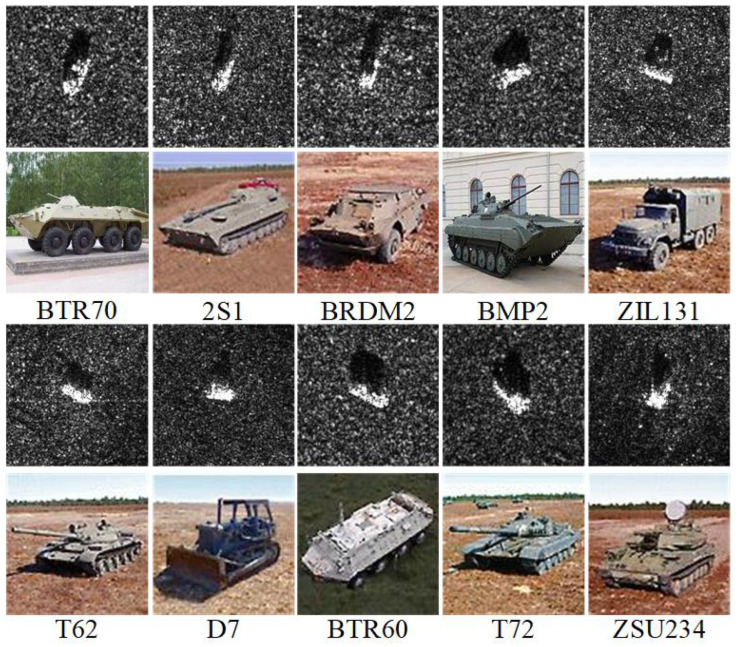
MSTAR dataset SAR and optical imagery.

Figure 6 illustrates the targets in both SAR and optical imagery. Table 1 details the dataset configuration, with 4 base classes and remaining classes distributed across incremental phases.

#### 4.1.2. SAR-AIRcraft-1.0 Dataset

The SAR-AIRcraft-1.0 dataset [22], from the Aerospace Information Research Institute, Chinese Academy of Sciences, contains high-resolution SAR images of commercial aircraft captured by the GaoFen-3 satellite in spotlight mode. It includes seven aircraft categories: A220, A320, A330, ARJ21, Boeing737, Boeing787, and others. Challenges include complex background clutter and fine-grained inter-class variations.

A single incremental configuration is used for SAR-AIRcraft-1.0:

- **Setting D**: 3 incremental phases (T=3), 1 new class per phase (S=1)

**Figure 7:**
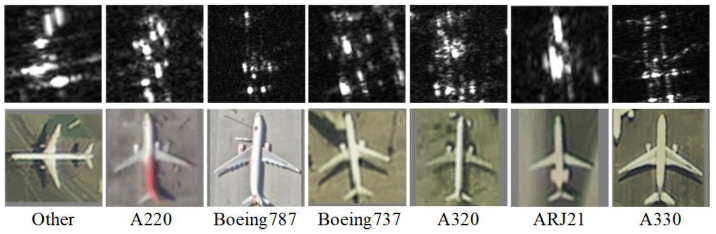
SAR-AIRcraft-1.0 Dataset SAR and optical imagery.

Figure 7 shows SAR and optical imagery of targets. Table 2 details the dataset configuration, with 4 base classes and 3 incrementally added classes.

### 4.2. Implementation Details

Our implementation follows a consistent protocol across all experiments. For the proposed MPFR method, we leverage both SAR amplitude images and complex-valued data when using the MSTAR dataset, while for SAR-AIRcraft-1.0, we utilize only SAR amplitude images due to data availability. All comparative methods employ a ResNet-18 backbone as the feature extractor with SAR amplitude images as input.

Input images are normalized to the range [0, 1] and resized to 128×128 pixels. All experiments are implemented using PyTorch (v1.12.1; Meta Platforms, Menlo Park, CA, USA) and executed on NVIDIA GeForce RTX 4090 GPUs (NVIDIA Corporation, Santa Clara, CA, USA). Models are trained for 100 epochs using the Adam optimizer ($\beta_{1}=0.9$, $\beta_{2}=0.999$) with a batch size of 128. The initial learning rate is set to 0.001 and decayed following a cosine annealing schedule. For our MPFR method, we set the number of prototypes per class to m=10 based on empirical analysis.

### 4.3. Evaluation Metrics

We employ two principal metrics to comprehensively evaluate class-incremental learning performance:

- **Average Accuracy (A)**: The mean top-1 classification accuracy across all incremental phases (including the base phase), providing an overall measure of learning capability:
  (18) $$A=\frac{1}{T+1}\sum_{t=0}^{T}A_{t}$$
  where *T* denotes the total number of incremental phases and $A_{t}$ represents the accuracy at phase *t*.
- **Forgetting Rate (FR)**: Quantifies the stability of knowledge retention by measuring performance degradation on base classes after completing all incremental phases:
  (19) $$\mathrm{FR}=(A_{0}-A_{T})\times 100\%$$
  where $A_{0}$ indicates the accuracy on base classes after initial training, and $A_{T}$ denotes the corresponding accuracy after all *T* incremental phases.

Both metrics are computed over three independent runs, and we report mean values with standard deviations to ensure statistical significance.

### 4.4. Comparative Evaluation

We conduct extensive comparative experiments against several state-of-the-art class-incremental learning methods, including FeTrIL [17], WA [37], SimpleCIL [18], iCaRL [36], LwF [35], ADC [39], and MIM-CIL [42].

To ensure a strictly fair comparison and isolate the contribution of the incremental learning strategies, all comparative methods are implemented using the identical ResNet-18 backbone network. By standardizing the feature extractor, we ensure that performance differences are solely attributable to the effectiveness of the incremental learning algorithms rather than discrepancies in model capacity. All methods are evaluated on both MSTAR [21] and SAR-AIRcraft-1.0 [22] datasets under identical experimental conditions with an input size of 128×128, the same data augmentation, and identical optimizer settings.

#### 4.4.1. Results on MSTAR Dataset

Table 3 presents the comprehensive comparison on the MSTAR dataset across three incremental configurations.

**Figure 8:**
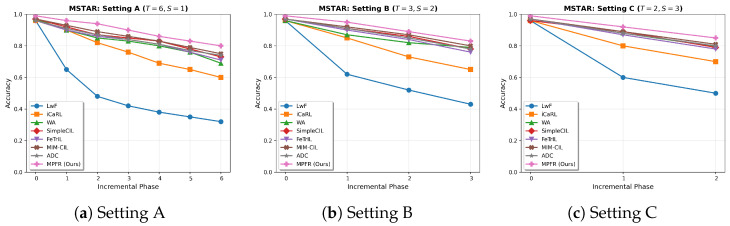
Accuracy trends across incremental phases on MSTAR dataset: (**a**) Setting A (T=6,S=1), (**b**) Setting B (T=3,S=2), (**c**) Setting C (T=2,S=3). Our MPFR method demonstrates superior performance with minimal accuracy degradation.

The experimental results reveal that our MPFR method achieves the highest average accuracy across all settings (e.g., 89.7% in Setting A), as shown in Figure 8, representing a 2.2% absolute improvement over MIM-CIL and a 3.3% improvement over ADC. This performance advantage can be attributed to the fundamental differences in how these methods model the SAR feature space.

Regarding SimpleCIL and FeTrIL, these methods typically rely on a single Gaussian prototype or mean vector to represent each class. As discussed in Section 3.4, SAR targets exhibit extreme aspect sensitivity, forming “disjointed blobs” in the feature space rather than a single compact cluster. Single-prototype methods average out these distinct modes, losing critical topological information. MPFR’s multi-center approach preserves these modes, leading to significantly lower forgetting rates (19.8% vs. 24.7% for SimpleCIL).

In comparison with MIM-CIL, while it effectively maximizes global mutual information between old and new tasks to reduce forgetting, it focuses on statistical dependency rather than geometric structure. In scenarios with large depression angle variations (MSTAR SOC), preserving the geometry of specific scattering topologies is crucial. MPFR explicitly reconstructs these geometric sub-structures via local clustering, providing a more fine-grained representation than MIM-CIL’s global optimization, hence the 2.2% accuracy gain.

Unlike ADC, which employs adversarial perturbations to estimate and compensate for feature drift, MPFR uses Gaussian diffusion based on actual stored centroids (μ). While ADC’s generated “drifted” samples are based on a learned adversarial direction, they may not fully capture the complex, non-linear scattering variations of radar targets. In contrast, MPFR ensures that the reconstructed features remain faithful to the original multi-modal distribution of the target, resulting in superior stability.

#### 4.4.2. Results on SAR-AIRcraft-1.0 Dataset

Table 4 shows the performance comparison on the more challenging SAR-AIRcraft-1.0 dataset.

On this challenging dataset, our method achieves an average accuracy of 92.3%, outperforming the second-best method (MIM-CIL) by 2.2% and reducing the forgetting rate by 3.1%. The SAR-AIRcraft-1.0 dataset contains complex background clutter and fine-grained inter-class variations (e.g., distinguishing Boeing 737 from 787). Methods like ADC and MIM-CIL may struggle to disentangle the target features from background noise when enforcing global constraints. MPFR’s advantage here lies in its ability to cluster features that share similar semantic content (aircraft structure) while potentially isolating outlier clusters caused by background variations. By reconstructing the distribution around these clean, multi-modal centers, MPFR maintains high discriminability even for fine-grained categories, demonstrating that geometric reconstruction is a robust strategy for complex real-world SAR imagery.

#### 4.4.3. Computational Efficiency Analysis

To evaluate the computational efficiency of the proposed method, we compared the training costs of MPFR against several existing approaches, including recent methods like ADC and MIM-CIL. To ensure a fair comparison, all methods were evaluated using the same ResNet-18 backbone on an NVIDIA RTX 4090 GPU. Table 5 details the average accuracy and training time per incremental phase on the MSTAR dataset under Setting A (T=6,S=1).

As shown in Table 5, regularization-based and replay-based methods (LwF, iCaRL, WA) incur higher computational overhead (2.6–3.5 min) due to gradient backpropagation. Similarly, ADC and MIM-CIL require significant computation (2.6 min and 2.9 min) for adversarial generation or mutual information optimization.

In contrast, our MPFR method achieves the highest accuracy (89.7%) with a training time of just 0.6 min. While SimpleCIL and FeTrIL are faster (0.2 min and 0.5 min), they suffer from notable performance drops compared to MPFR (e.g., −4.7% for SimpleCIL and −6.3% for FeTrIL). MPFR effectively balances the trade-off, offering superior performance with a highly efficient update speed suitable for real-time applications.

#### 4.4.4. Cross-Dataset Step-Wise Incremental Learning

To robustly evaluate the generalizability of MPFR under severe domain shifts and continuous adaptation scenarios, we designed a rigorous Cross-Dataset Step-wise Incremental Learning experiment (Setting E). Unlike standard settings where the domain remains constant, this scenario requires the model—initially trained on ground targets (MSTAR)—to progressively learn aerial targets (SAR-AIRcraft-1.0) in multiple steps.

**Setting E Configuration (Ground-to-Air Step-wise Transfer):**

- **Base Phase:** All 10 classes from the MSTAR dataset (Ground Vehicles).
- **Incremental Phases (T=3):** The 7 classes from SAR-AIRcraft-1.0 are added sequentially over 3 phases to simulate a continuous stream of new aerial threats:
  - **Phase 1:** 3 classes (A220, A320, A330).
  - **Phase 2:** 2 classes (ARJ21, Boeing737).
  - **Phase 3:** 2 classes (Boeing787, Other).

This setting is particularly challenging because the feature extractor, frozen after learning ground vehicle features, must accommodate aircraft features that possess significantly different scattering structures and background clutter distributions.

Table 6 summarizes the performance. We report the Average Accuracy (*A*) across all phases (Base + 3 Incremental phases) and the Final Forgetting Rate (FR) of the base MSTAR classes after the final phase.

As illustrated in Table 6, MPFR outperforms all comparative methods in this cross-domain continuous learning scenario. Standard replay-free methods like SimpleCIL and FeTrIL experience performance drops because their single-prototype representations struggle to adapt to the “alien” distribution of aircraft targets when the backbone is frozen on ground vehicle data. The domain shift causes the new aircraft features to be loosely distributed, which single centroids fail to capture accurately, leading to overlap with old classes.

In contrast, MPFR’s multi-center strategy flexibly models the dispersed scattering features of the new aerial targets as multiple sub-clusters. This capability allows the model to find “open spaces” in the embedding space for new classes without disrupting the decision boundaries of the existing MSTAR classes. The low forgetting rate (5.8%) confirms that MPFR effectively isolates the new domain knowledge from the old, making it highly suitable for real-world multi-domain recognition systems.

### 4.5. Ablation Studies

To quantitatively assess the individual contributions of each proposed component and verify the robustness of our framework, we conduct comprehensive ablation studies on the MSTAR dataset using the Setting A (T=6,S=1) configuration.

#### 4.5.1. Multi-Scale Hybrid Attention and Training Strategy

We first evaluate the effectiveness of the Multi-scale Hybrid Attention (MHA) feature fusion module and the joint supervision training strategy. Table 7 presents the comparative results of different feature fusion approaches and training strategies.

The results demonstrate the progressive improvement achieved by each component. The integration of ASC features with SAR amplitude images provides a 3.2% accuracy improvement over using SAR data alone, confirming the value of complementary electromagnetic scattering characteristics. The MHA fusion mechanism further enhances performance by 2.2% compared to simple concatenation, highlighting the importance of multi-scale processing and adaptive feature weighting. Most significantly, the joint supervision strategy provides an additional 4.1% improvement, validating that the combination of supervised contrastive loss and additive angular margin loss effectively enhances feature discriminability and robustness for incremental learning.

#### 4.5.2. Multi-Center Prototype Strategy

We further investigate the effectiveness of the multi-center prototype strategy compared to single-center approaches, with particular attention to the impact of prototype quantity on performance and storage efficiency. Table 8 presents the quantitative results.

The multi-center prototype approach consistently outperforms the single-center baseline, with the m=10 configuration achieving optimal performance (89.7% average accuracy, 19.8% forgetting rate). This represents a substantial 5.8% improvement in accuracy and 15.8% reduction in forgetting rate compared to the single-prototype approach.

A single prototype forces the assumption of unimodal Gaussian distribution, which severely underfits the true feature manifold of SAR targets that exhibit significant aspect-dependent scattering. Our multi-center approach addresses this by approximating the complex distribution through a Gaussian Mixture Model. The performance improvement from m=1 to m=10 demonstrates that increasing prototype diversity effectively captures the underlying data structure. However, the slight performance degradation at m=20 suggests the onset of over-clustering, where the model begins to fit noise rather than meaningful semantic modes. The choice of m=10 represents an optimal balance between performance and storage efficiency.

#### 4.5.3. Impact of Feature Extractor Backbone

To verify the generalization capability of the MPFR framework and ensure that its performance is not strictly dependent on the ResNet-18 architecture, we evaluated our method using various feature extractor backbones with different parameter scales. Specifically, we compared the lightweight ResNet-18 with deeper Convolutional Neural Networks (VGG-16 [48], ResNet-50 [49]) and Vision Transformer architectures (ViT-S/16 and ViT-B/16 [50]). For ViT models, we utilized weights pre-trained on ImageNet and fine-tuned them on the base classes to accommodate the SAR data scale. All experiments were conducted under the MSTAR Setting A (T=6,S=1) configuration.

Table 9 summarizes the performance and model size (number of parameters) across different architectures.

The results indicate that the MPFR framework is architecture-agnostic and functions effectively across different types of backbones. Regarding the efficiency of Residual Networks, despite having the fewest parameters (11.7 M), ResNet-18 achieves competitive performance (89.7%), outperforming the much larger VGG-16 (138.4 M). This justifies our choice of ResNet-18 as the default backbone for resource-constrained onboard SAR applications.

When comparing CNNs with Transformers of similar size, ViT-S/16 (22.1 M) outperforms ResNet-50 (25.6 M) by 0.4% in accuracy and reduces the forgetting rate by 0.4%. This suggests that the Transformer architecture provides highly discriminative feature representations for SAR targets, which effectively complements our multi-center prototype reconstruction. In terms of scalability, scaling up to ViT-B/16 (86.6 M) results in the highest average accuracy (91.2%). However, the marginal gain over ViT-S (0.3%) comes at a cost of nearly 4× the parameters, highlighting ViT-S as a highly efficient alternative for high-performance scenarios.

Despite the superior performance of ViT architectures, we retained ResNet-18 as the default backbone in our main experiments to ensure a fair comparison with existing baselines (e.g., FeTrIL, SimpleCIL), which predominantly report results using ResNet-18.

## 5. Conclusions

In this paper, we proposed a novel Class-Incremental Learning method named Multi-center Prototype Feature Distribution Reconstruction (MPFR) to address the challenge of catastrophic forgetting in SAR ATR systems. Recognizing that standard single-prototype approaches fail to capture the complex, multi-modal feature manifolds caused by the aspect-sensitivity of SAR targets, MPFR introduces a robust mechanism to model and reconstruct these distributions without storing raw historical data.

Our method rests on two pillars: a high-performance feature extraction module and a distribution reconstruction mechanism. The Multi-scale Hybrid Attention network, optimized via a joint supervision strategy comprising Supervised Contrastive Loss and Additive Angular Margin Loss, successfully fuses SAR amplitude information with physically meaningful Attributed Scattering Centers. This ensures the learned embeddings are both compact and discriminative. Furthermore, the Multi-center Prototype strategy approximates the feature space of old classes using multiple cluster centroids and Gaussian diffusion. This allows the classifier to retain knowledge of the topological structure of historical data while adapting to new categories.

Extensive experiments on the MSTAR and SAR-AIRcraft-1.0 datasets validated the superiority of MPFR. It consistently outperformed state-of-the-art methods, including recent SAR-specific CIL approaches and general CIL frameworks, achieving up to 92.3% average accuracy with minimal forgetting. Ablation studies further confirmed that multi-center modeling is critical for handling the high intra-class variance inherent in radar imagery.

Future work will focus on three key directions. First, to address the challenge of hyperparameter selection noted in the ablation studies, we plan to investigate adaptive clustering mechanisms to automatically determine the optimal number of centroids per class based on the complexity of their feature distributions. Second, building on the promising results from the cross-dataset experiments, we aim to further enhance the model’s robustness against severe domain shifts and speckle noise, exploring its applicability in more complex cross-sensor incremental learning scenarios to support multi-platform cooperative reconnaissance. Finally, we will extend the framework to Open-Set Recognition (OSR) settings, enabling the system to not only incrementally learn new classes but also effectively detect and reject unknown targets in dynamic operational environments.

## Figures and Tables

**Table 1:** MSTAR dataset configurations for class-incremental learning.

Setting	Phase	Class	Training Samples	Test Samples
A	Base	2S1, BMP2, BRDM2, BTR60	1086	938
	Phase 1	BTR70	233	196
	Phase 2	D7	299	274
	Phase 3	T62	299	273
	Phase 4	T72	232	196
	Phase 5	ZIL131	299	274
	Phase 6	ZSU234	299	274
B	Base	2S1, BMP2, BRDM2, BTR60	1086	938
	Phase 1	BTR70, D7	532	470
	Phase 2	T62, T72	531	469
	Phase 3	ZIL131, ZSU234	598	548
C	Base	2S1, BMP2, BRDM2, BTR60	1086	938
	Phase 1	BTR70, D7, T62	831	743
	Phase 2	T72, ZIL131, ZSU234	830	744
**Total**	**10 classes**		**2747**	**2425**

**Table 2:** SAR-AIRcraft-1.0 dataset configuration for class-incremental learning.

Phase	Class Names	Training Samples	Test Samples
Base	A220, A320, A330, ARJ21	4000	800
Phase 1	Boeing737	1000	200
Phase 2	Boeing787	1000	200
Phase 3	Other	1000	200
**Total**	**7 classes**	**7000**	**1400**

**Table 3:** Comparative results on MSTAR dataset under different incremental configurations.

	Setting A		Setting B		Setting C	
**Method**	**A(%)**	**FR (%)**	**A(%)**	**FR (%)**	**A(%)**	**FR (%)**
LwF	59.4 ± 1.5	64.2 ± 2.1	63.3 ± 1.3	53.5 ± 1.8	68.7 ± 1.1	46.3 ± 1.5
iCaRL	77.3 ± 1.2	36.4 ± 1.7	79.8 ± 1.0	31.2 ± 1.4	82.0 ± 0.9	26.1 ± 1.2
WA	82.9 ± 1.0	28.3 ± 1.4	86.0 ± 0.8	17.6 ± 1.1	88.2 ± 0.7	17.8 ± 1.0
SimpleCIL	85.0 ± 0.8	24.7 ± 1.2	88.0 ± 0.6	19.2 ± 0.9	88.0 ± 0.5	17.5 ± 0.8
FeTrIL	83.4 ± 0.6	25.3 ± 1.0	86.8 ± 0.5	21.4 ± 0.7	87.3 ± 0.4	19.6 ± 0.6
ADC	86.4 ± 0.8	22.8 ± 1.3	88.6 ± 0.7	19.1 ± 1.0	89.1 ± 0.6	17.1 ± 0.8
MIM-CIL	87.5 ± 0.7	21.3 ± 1.1	89.2 ± 0.6	18.5 ± 0.9	89.8 ± 0.5	16.2 ± 0.7
**MPFR (Ours)**	**89.7 ± 0.4**	**19.8 ± 0.7**	**91.5 ± 0.3**	**16.7 ± 0.5**	**92.0 ± 0.3**	**14.9 ± 0.4**

**Table 4:** Comparative results on SAR-AIRcraft-1.0 dataset (Setting D: T=3,S=1).

Method	A (%)	FR (%)
LwF	72.3 ± 1.6	41.7 ± 2.3
iCaRL	83.3 ± 1.3	24.5 ± 2.0
WA	87.3 ± 1.1	19.3 ± 1.7
SimpleCIL	88.0 ± 0.9	17.8 ± 1.4
FeTrIL	88.3 ± 0.7	17.6 ± 1.2
ADC	89.4 ± 0.8	15.2 ± 1.3
MIM-CIL	90.1 ± 0.6	13.5 ± 1.1
**MPFR (Ours)**	**92.3 ± 0.5**	**10.4 ± 1.0**

**Table 5:** Comparison of computational efficiency and performance on MSTAR dataset (Setting A). Training time represents the average duration per incremental phase.

Method	Avg. Accuracy A (%)	Avg. Train Time (min)
LwF	59.4	3.5
iCaRL	77.3	2.8
WA	82.9	2.6
SimpleCIL	85.0	0.2
FeTrIL	83.4	0.5
ADC	86.4	2.6
MIM-CIL	87.5	2.9
**MPFR (Ours)**	**89.7**	**0.6**

**Table 6:** Comparative results of Cross-Dataset Step-wise Incremental Learning (Setting E). The model learns 10 MSTAR classes followed by 7 SAR-AIRcraft classes in 3 steps (10→3→2→2).

Method	Average Accuracy A(%)	Base Forgetting Rate FR (%)
LwF	61.5 ± 1.9	48.2 ± 2.4
iCaRL	75.2 ± 1.6	30.5 ± 2.1
WA	85.4 ± 1.3	12.7 ± 1.8
SimpleCIL	82.6 ± 1.0	14.3 ± 1.2
FeTrIL	83.1 ± 0.9	13.8 ± 1.1
ADC	85.9 ± 1.1	11.2 ± 1.4
MIM-CIL	86.5 ± 0.8	10.1 ± 1.0
**MPFR (Ours)**	**91.2 ± 0.6**	**5.8 ± 0.9**

**Table 7:** Ablation study on MHA feature fusion and training strategy.

Method	A(%)	FR (%)
SAR only (CE Loss)	76.9 ± 1.2	67.3 ± 1.8
SAR + ASC (Simple Concatenation)	80.1 ± 0.8	58.4 ± 1.3
SAR + ASC (MHA w/o Attention)	82.3 ± 0.7	49.2 ± 1.1
SAR + ASC (MHA, CE Loss)	85.6 ± 0.6	32.7 ± 0.9
**SAR + ASC (MHA, Joint Loss)**	**89.7 ± 0.4**	**19.8 ± 0.7**

**Table 8:** Ablation study on multi-center prototype strategy.

Prototype Strategy	A(%)	FR (%)	Storage (KB)
Single-center (m=1)	83.9 ± 0.8	35.6 ± 1.3	20.5
Multi-center (m=5)	87.3 ± 0.6	24.8 ± 1.0	102.4
**Multi-center (m=10)**	**89.7 ± 0.4**	**19.8 ± 0.7**	**204.8**
Multi-center (m=15)	89.5 ± 0.4	20.0 ± 0.7	307.3
Multi-center (m=20)	89.1 ± 0.5	20.3 ± 0.8	409.6

**Table 9:** Performance comparison of MPFR using different feature extractor backbones (MSTAR Setting A).

Backbone	Type	Params (M)	A(%)	FR (%)
VGG-16	CNN (Deep)	138.4	88.9 ± 0.6	20.5 ± 0.9
ResNet-18 (Default)	CNN (Residual)	11.7	89.7 ± 0.4	19.8 ± 0.7
ResNet-50	CNN (Deep Residual)	25.6	90.5 ± 0.4	18.9 ± 0.6
ViT-S/16	Transformer (Small)	22.1	90.9 ± 0.5	18.5 ± 0.7
ViT-B/16	Transformer (Base)	86.6	91.2 ± 0.5	18.2 ± 0.8

## Data Availability

Publicly available datasets were analyzed in this study. The MSTAR dataset can be found in reference [21]. The SAR-AIRcraft-1.0 dataset is openly available in the *Journal of Radars* repository at https://radars.ac.cn/web/data/getData?dataType=SAR-Airport accessed on 29 September 2025 or reference [22].
